# Susceptibility of *Anopheles gambiae *and *Anopheles stephensi *to tropical isolates of *Plasmodium falciparum*

**DOI:** 10.1186/1475-2875-6-139

**Published:** 2007-10-24

**Authors:** Jennifer CC Hume, Mark Tunnicliff, Lisa C Ranford-Cartwright, Karen P Day

**Affiliations:** 1 Peter Medawar Building for Pathogen Research and Department of Zoology, South Park road, University of Oxford, Oxford OX1 3SY, UK; 2 Division of Infection and Immunity, Glasgow Biomedical Research Centre, University of Glasgow, 120 University Place, Glasgow G12 8TA, UK; 3 Laboratory of Malaria and Vector Research, NIH/NIAID, 12735 Twinbrook Parkway, Rockville, MD 20892, USA; 4 Department of Medical Parasitology, NYU School of Medicine, 341 East 25^th ^Street, New York, NY 10010, USA

## Abstract

**Background:**

The susceptibility of anopheline mosquito species to *Plasmodium *infection is known to be variable with some mosquitoes more permissive to infection than others. Little work, however, has been carried out investigating the susceptibility of major malaria vectors to geographically diverse tropical isolates of *Plasmodium falciparum *aside from examining the possibility of infection extending its range from tropical regions into more temperate zones.

**Methods:**

This study investigates the susceptibility of two major tropical mosquito hosts (*Anopheles gambiae *and *Anopheles stephensi*) to *P. falciparum *isolates of different tropical geographical origins. Cultured parasite isolates were fed via membrane feeders simultaneously to both mosquito species and the resulting mosquito infections were compared.

**Results:**

Infection prevalence was variable with African parasites equally successful in both mosquito species, Thai parasites significantly more successful in *An. stephensi*, and PNG parasites largely unsuccessful in both species.

**Conclusion:**

Infection success of *P. falciparum *was variable according to geographical origin of both the parasite and the mosquito. Data presented raise the possibility that local adaptation of tropical parasites and mosquitoes has a role to play in limiting gene flow between allopatric parasite populations.

## Background

The susceptibility of *Anopheles *mosquitoes to *Plasmodium *infection is defined by the ability of a mosquito vector to support parasite development from gamete fertilization through to sporozoite production. This susceptibility ranges from complete receptiveness, where all individuals support infection, to the opposite end of the spectrum, total refractoriness, where no individuals support infection. The majority of mosquito vectors are positioned somewhere between the two extremes depending on geographical origin of both the parasite and the mosquito [1-10]. In essence, there is a degree of compatibility that exists between parasite and mosquito and the extent of this compatibility determines the successful transmission of infection.

Theoretical co-evolutionary models propose that parasites are locally adapted to their hosts and that parasite fitness decreases as geographical distance from the host increases [11]. Thus, *Plasmodium falciparum *parasites would be expected to transmit more successfully through a local, indigenous, mosquito species rather than a non-local species. Local adaptation in *Plasmodium *was investigated previously during the era of malaria eradication, when experiments largely focused on the susceptibility of *Anopheles *mosquitoes from non-malarious areas to tropical *P. falciparum *parasites. In particular, the re-introduction of malaria to Europe was examined, leading to the discovery that indigenous European mosquitoes are almost completely refractory to tropical *P. falciparum *infection [1,3-5]. In contrast, work carried out in North America demonstrated that the indigenous vector, *Anopheles freeborni*, is highly susceptible to many species of *Plasmodium *from widely separated geographical areas (reviewed [12]).

Contemporary patterns of human migration provide numerous occasions for allopatric parasite populations to interbreed allowing the transference of novel phenotypes, such as drug resistance, to new parasite populations both within and between continents. A better understanding of the limitations imposed by mosquito susceptibility would be advantageous in predicting the potential spread of these undesirable phenotypes and allow cost-effective vector control measures to be implemented. This paper aims to build on previous investigations and examine the compatibility between parasite and host by examining the transmission of tropical *P. falciparum *parasites through non-indigenous tropical vectors. Indeed an extensive survey of the literature reveals that this is the first published report describing the attempted challenge of the African vector *Anopheles gambiae *with Thai and Papua New Guinean (PNG) *P. falciparum *parasites.

## Methods

### Mosquitoes

To ensure that parasite-mosquito compatibility was correctly identified, paired feeding of both a control and an experimental mosquito species was undertaken. Ideally the control species should correspond to the indigenous mosquito vector and indeed for the African parasites this was the case with *An. gambiae *selected. For the Thai and PNG parasites however, *Anopheles stephensi *was selected rather than *Anopheles dirus *and *Anopheles farauti*. The problematic nature of rearing *An. dirus *and *An. farauti *was the main reason behind this decision and was supported by a previous experiment indicating that Malayan *P. falciparum *parasites transmit successfully through *An. stephensi *[13].

*An. gambiae *(G3 strain) and *An. stephensi *(sd500 strain) colonies were reared from eggs obtained from Imperial College (*An. stephensi *and *An. gambiae*) and the London School of Hygiene and Tropical Medicine (*An. gambiae*). Colonies were maintained as described by Bell and Ranford-Cartwright [14]. Adult mosquitoes were fed 5% glucose in 0.05% para-aminobenzoic acid water.

### Parasites

Ten uncloned parasite isolates from three different geographical locations were used: P018, P019, and P020 from Thailand; K39, M24 from Kenya; PNG12.2, PNG37.2, PNG51.1, 1776.3.7 from PNG. In addition, the well-characterized lab clone 3D7 was used. *In vitro *parasite cultures were maintained in RPMI 1640 (25 mM HEPES, 2 mM L-glutamine, 300 mM hypoxanthine) supplemented with 25 mM sodium bicarbonate and 10% human serum (from donors residing in non-malarious regions), in an atmosphere of 5% CO_2_, 5% O_2_, and 90% N_2_. Cells were subcultured into O, Rh^+ ^erythrocytes. Gametocyte cultures were initiated at 5% haematocrit and 1% parasitaemia (mixed stages) and maintained for up to 17 days.

### Membrane feeding assay

Non-bloodfed adult female mosquitoes 4–7 days post-emergence were collected from stock cages and placed into mesh-topped, wax-lined pots 1 day prior to the feed. Up to 100 mosquitoes were contained within each pot and they were provided with only distilled water for 24 hrs prior to bloodfeeding. Two gametocyte cultures, 14 and 17 days old, were mixed and diluted to 0.5–1% gametocytaemia with an erythrocyte/serum mixture at 40% haematocrit as described by Carter and others [15]. This infective feed was fed to both mosquito species simultaneously using an artificial feeder (Hemotek^® ^membrane feeding system) and mosquitoes were allowed to feed for 20 minutes through parafilm. A sample of the infective feed was examined under light microscopy and exflagellation was recorded. Unfed mosquitoes were removed a couple of hours post-feeding. Mosquitoes were maintained for 10 days at 28°C, 70–80% humidity and provided with 5% glucose solution in PABA water. Midgut dissection was performed on day 10 post-feeding with midguts examined under light microscopy for the presence of oocysts. The number of times each parasite isolate was fed to mosquito combinations was as follows: 3D7, 3 paired feeds; K39, 2 paired feeds, 1 single feed *An. stephensi*; M24, 2 paired feeds; P018, 2 paired feeds, P019, 2 paired feeds; P020, 1 paired feed; PNG12.2, 3 single feeds *An. stephensi*; PNG37.2, 1 paired feed; PNG51.1, 1 paired feed; 1776.3.7, 1 paired feed. Although a 3D7 feed was not included as a positive mosquito control, multiple parasite isolates were routinely fed to mosquitoes at the same time point. Commonly, these isolates produced different infection outcomes in the same mosquito species implying that mosquito receptivity did not noticeably vary or diminish over the course of these experiments producing false negative results.

### Data analysis

χ^2 ^comparisons were performed to compare infection prevalence of each parasite isolate between the two mosquito species (*An. stephensi *Vs. *An. gambiae*). Oocysts were not distributed normally but rather fitted a negative binomial distribution making it inappropriate to use mean-based parametric statistics in analysis [16]. Accordingly, oocyst burdens between infected mosquitoes were compared for each isolate using Mann-Whitney tests (*An. stephensi *Vs. *An. gambiae*). Statistical analyses were performed using SPSS 11.0 and statistical differences were considered significant at a probability of *P *< 0.01.

## Results

1,197 mosquitoes were dissected and examined; 403 (33.7%) were infected with *P. falciparum*. Infection rate was 33% (230 of 696) for *An. stephensi *and 34.5% (173 of 501) for *An. gambiae*. Ten *P. falciparum *isolates from three geographical origins were used to evaluate the differential susceptibility of *An. stephensi *and *An. gambiae *to tropical parasites (Table 1). Variation in the infection rate between *P. falciparum *isolates was clearly observed with a number of parasite isolates failing to infect one or both mosquito species. In particular, the isolates PNG12.2 and PNG51.1 from PNG appeared to be non-infectious producing no oocysts in either mosquito species. Similarly, PNG37.2, 1776.3.7, and M24 produced a very limited number of mosquito infections suggesting that they are only minimally infective. The remaining five parasite isolates appeared to be highly infective and produced some surprising results. The African parasite K39, and 3D7, infected both *An. gambiae *and *An. stephensi *equally (*P *> 0.01) although the actual infection prevalence demonstrated by the two isolates was markedly different, with K39 infection levels three times higher than 3D7.

**Table 1:** Infection prevalence of different *Plasmodium falciparum *isolates in two mosquito species: *Anopheles stephensi *and *Anopheles gambiae*

		***Anopheles stephensi***		***Anopheles gambiae***		
			
**Geographical location**	**Parasite isolate**	**No. infected/dissected**	**% infected**	**No. infected/dissected**	**% infected**	***P *value**
**?**	3D7	32/136	24	19/108	18	0.257
**Africa**	K39	89/119	75	117/136	86	0.023
	M24	0/78	0	2/32	6	0.083
**Thailand**	P018	32/42	76	14/38	37	<0.001*
	P019	60/78	77	14/66	21	<0.001*
	P020	11/24	46	6/41	15	<0.001*
**Papua New Guinea**	PNG12.2	0/117	0	-	-	N/A
	PNG37.2	2/31	7	0/21	0	0.35
	PNG51.1	0/31	0	0/41	0	N/A
	1776.3.7	4/40	10	1/18	6	0.577

Values represent total pooled experimental results with prevalence's expressed as percentages (no. of mosquitoes infected/no. of mosquitoes dissected). Number of times each parasite isolate was fed to mosquito combinations was as follows: 3D7, 3 paired feeds; K39, 2 paired feeds, 1 single feed *An. stephensi*; M24, 2 paired feeds; P018, 2 paired feeds, P019, 2 paired feeds; P020, 1 paired feed; PNG12.2, 3 single feeds *An. stephensi*; PNG37.2, 1 paired feed; PNG51.1, 1 paired feed; 1776.3.7, 1 paired feed. * Indicates statistically significant comparison performed by χ^2 ^test; probability considered significant at *P *< 0.01. N/A = not applicable.

The Thailand parasites provided the most interesting results with all three Thai isolates demonstrating significant differences (*P *< 0.001) in infection prevalence between the two mosquito species. P018 and P019 infected *An. stephensi *at a comparable level to K39 but unlike K39, this prevalence diminished sharply in *An. gambiae*. Similarly, P020 infectivity was significantly reduced in *An. gambiae *but it also displayed a lower prevalence in *An. stephensi*, than P018 and P019.

Amongst infected mosquitoes, the intensity of infection was variable both across different parasite isolates and between mosquito species (Table 2). Median oocyst number per infected midgut varied from 18 (K39) to 1 (1776.3.7) with similar variations in 25–75 percentiles ranging from 1–46 (K39) to 1-1 (1776.3.7). As with prevalence, K39 was shown to produce the highest infection intensities but neither K39 nor 3D7 displayed any difference in oocyst numbers between mosquito species. Unlike infection prevalence, only one Thai isolate demonstrated a statistically significant difference in infection intensity; P019 produced significantly more oocysts per infected *An. stephensi *mosquito than *An. gambiae *(*P *< 0.001). Neither of the other Thai isolates displayed this trend but this appears to be due to a lower intensity of infection in *An. stephensi *rather than an increase of infection intensity in *An. gambiae*.

**Table 2:** Intensity of infection of different *Plasmodium falciparum *isolates in paired feeds to *Anopheles stephensi *and *Anopheles gambiae*

**Parasite isolate**	**Median number of oocysts per infected midgut (25–75 percentile)**		***P *value**
		
	*Anopheles stephensi*	*Anopheles gambiae*	
3D7	1.5 (1–2)	2 (1–3)	0.251
K39	12 (1–46)	18 (6–46)	0.343
M24	N/A: no infection	1 (N/A: too few infections)	-
P018	3 (1–9)	3 (1–4)	0.297
P019	10 (2–41)	2 (1–2)	< 0.001*
P020	2 (2–4)	2 (1–2)	0.711
PNG12.2	N/A: no infection	-	-
PNG37.2	N/A: too few infections	N/A: no infection	-
PNG51.1	N/A: no infection	N/A: no infection	-
1776.3.7	1 (1-1)	1 (N/A: too few infections)	0.8

* Indicates statistically significant comparison performed by Mann Whitney test; probability considered significant at *P *< 0.01. N/A = not applicable

## Discussion

Population-based data on microsatellites [17], mitochondrial DNA haplotypes [18], and chromosome-wide SNP haplotypes [19] reveal that *P. falciparum *has a well-differentiated population structure demonstrating strong clustering according to geographical origin. Whether this differentiation is solely due to geographical separation or indicative of a biological barrier remains an open question. Geographical separation is assumed, but in an era when modern transportation has led to a global community, it seems improbable that parasite populations stay differentiated by geography alone. The data presented in this paper clearly demonstrate significant differences in transmission success of parasites according to geographical origin of the mosquito and it seems apposite to propose a role for the vector in facilitating population separation.

The infectivity of 3D7 in this study was visibly lower than previous studies [14], but crucially, the reduction in infectivity was observed in both mosquito species and thus judged insignificant to the overall conclusions. Without knowing the geographical origins of 3D7, it is impossible to determine which mosquito species would be the indigenous transmission vector and indeed 3D7 demonstrates no significant differences, either in prevalence or infection intensity, between the two mosquito vectors. The African isolate, K39, also demonstrates the ability to infect both mosquito species equally successfully, and it can be hypothesized that this may be due to maintenance of ancestral genetic diversity or, as recently suggested by Sinden and colleagues, that a longer period of insect-parasite co-evolution may lead to an increase in parasite strategies against the insect immune system such that ancestral strains could be expected to be transmitted by a broader range of mosquito species [20].

In all the Thai parasites examined, infection prevalence was significantly reduced in *An. gambiae*. This trend was not, however, borne out in infection intensity with only P019 demonstrating a significant reduction in oocyst numbers in infected *An. gambiae *mosquitoes. While these observations are interesting and provide an insight into parasite-vector transmission dynamics, it is important to note that no parasite-mosquito combination produced total refractoriness as observed previously in other compatibility studies [1,3-5]. Unlike some studies, these experiments used laboratory maintained colonies of mosquitoes rather than wild-caught populations. Laboratory-maintained mosquito colonies are highly inbred and consequently can demonstrate markedly reduced microsatellite DNA polymorphism and heterozygosity [21], which may make them genetically dissimilar to the originally sampled population but whether they are representative of wild-caught populations in regards to transmission potential remains to be determined. Natural mosquito populations have been shown to demonstrate marked differences to *P. falciparum *infection with resistance to infection occurring at relatively high frequencies [22], thus, wider ranges in infection prevalence may be observed in the more diverse field populations. These preliminary experiments do demonstrate the potential for important differences in transmission potential of Thai compared to African parasites within *An. gambiae*, however, repetition of these results using wild-caught F1 reared progeny would be valuable.

PNG infection results ranged from zero to low infection among the four gametocyte-producing isolates tested in both mosquito species; PNG37.2 and 1776.3.7 produced successful infections. Poor exflagellation (1–2 exflagellation events in 20 fields) was observed in all the PNG isolates except 1776.3.7. Although this is far from an assured method of determining successful infection outcome, it does serve as an indication of the culture infectivity. Exflagellation of 1776.3.7 was excellent (>5 exflagellation events per field), comparable to K39, yet only 10% of *An. stephensi *mosquitoes were infected. Similarly, gametocyte production in these parasite isolates was comparable to the Thai and African parasites and there were no discernable microscopic distinctions regarding gametocyte health. This study represents the first published observations regarding PNG parasite isolates infecting *An. stephensi *or *An. gambiae *and it may be that parasites from this region do not transmit successfully through these non-indigenous mosquito species. Paired feedings of PNG parasites to *An. stephensi*/*An. gambiae *concurrent with the native PNG mosquito vector, *An. farauti*, are essential to determine true parasite-mosquito incompatibilities and determine the validity of these results. Interestingly, recent investigations have shown that *An. farauti *is only barely susceptible to *P. falciparum *strains from South America and Africa, with the respective authors urging the need to study co-indigenous parasite-mosquito combinations in this region [8,10].

A high level of parasite-mosquito compatibility in *An. farauti *may explain the low infection success of PNG parasite isolates in these studies, but the same can not be said for M24. Only two mosquitoes were positive for this isolate and both had a single oocyst. It appears that this parasite isolate displays very limited infectivity which is unrelated to geographical origin of the mosquito.

Studying the local adaptation and compatibility of *P. falciparum *isolates to different mosquito species is of use regarding gene transfer between allopatric parasite populations. Drug resistance is a burgeoning problem in malaria and global transportation links allow parasites to be rapidly transported between malarious areas. People within areas of intense malaria transmission habitually contain multiple parasite clones that are able to cross and recombine within the mosquito, leading to novel genotypes. In this manner, drug resistance genes can spread through populations. The spread of chloroquine resistance is well documented and is thought to have arisen independently in four different geographical areas – Southeast Asia, twice in South America and later in PNG [23]. Reports from Thailand suggest that resistance first occurred in the late 1950s but the subsequent spread into neighbouring areas, such as India, did not occur for 10–15 years. It appears that the spread of resistance within the limits of *Anopheles balabacensis *s.l. was fairly rapid but outside the confines of this permissive vector complex, progress was markedly slower. This could simply be due to the efficiency of the *An. balabacensis *complex but, as Wernsdorfer suggested [24], the possibility of a parasite-vector incompatibility reducing transmission success cannot be discounted.

## Conclusion

Variation in infection success of different *P. falciparum *isolates in two tropical mosquito species was clearly demonstrated. Local adaptation of the major tropical vectors to sympatric isolates could be important in shaping the parasite population structure by limiting gene flow and consequently have important implications in the fight against drug resistance. Further investigations with more isolates and extending the geographical range of parasites and mosquitoes can only help clarify the situation and provide further insights into global parasite-vector transmission dynamics.

## Authors' contributions

JCCH and KPD conceived and designed the study. JCCH conducted the experimental work with assistance from MT and LRC and supervision from KPD. JCCH drafted the manuscript with KPD. All authors read and approved the final manuscript.
